# Preparation and Properties Study of Wood-Based Cushioning Materials

**DOI:** 10.3390/polym15061417

**Published:** 2023-03-13

**Authors:** Shuang Pei, Zongying Fu, Jinsheng Gou, Yun Lu

**Affiliations:** 1 Key Laboratory of Wood Material Science and Application, Beijing Forestry University, Ministry of Education, Beijing 100083, China; 2 Beijing Key Laboratory of Wood Science and Engineering, Beijing Forestry University, Beijing 100083, China; 3 Research Institute of Wood Industry, Chinese Academy of Forestry, Beijing 100091, China

**Keywords:** elastic wood, cushioning qualities, mechanical properties, electromagnetic shielding

## Abstract

Traditional cushioning package materials, such as Expended Polystyrene (EPS) and Expanded Polyethylene (EPE), were made with petroleum-based plastics, which are harmful to the environment. It is crucial to develop renewable bio-based cushioning materials that can replace the aforementioned foams due to the rising energy demands of human society and the depletion of fossil fuels. Herein, we report an effective strategy for creating anisotropic elastic wood with special spring-like lamellar structures. Selective removal of lignin and hemicellulose by simple chemical treatment and thermal treatment of the samples after freeze-drying results in an elastic material with good mechanical properties. The resulting elastic wood has a reversible compression rate of 60% and a high elastic recovery (99% height retention after 100 cycles at 60% strain). Drop tests revealed that the elastic wood has excellent cushioning properties. In addition, the chemical and thermal treatments also enlarge the pores in the material, which is favorable for subsequent functionalization. By loading the elastic wood with a muti-walled carbon nanotube (MWCNT), electromagnetic shielding properties are achieved, while the mechanical properties of elastic wood remain unchanged. Electromagnetic shielding materials can effectively suppress various electromagnetic waves propagating through space and the resulting electromagnetic interference and electromagnetic radiation, improve the electromagnetic compatibility of electronic systems and electronic equipment, and ensure the safety of information.

## 1. Introduction

Packaging is an integral part of the distribution of goods. To protect the goods during logistics and transportation, not only external packaging but also internal cushioning material is required [1]. Commonly used foaming materials for cushioning packaging are organic foams, plant fiber foam material [2,3], metal foams [4,5,6,7], and ceramic foams [8,9]. Organic foams are closed-cell foams such as EPE, EPS, etc., which are made from organic materials and emulsifiers, foaming agents, etc. They are widely used due to their low density, excellent cushioning properties and low cost. However, they are non-biodegradable, thus causing “white pollution”, and are harmful to humans [10,11]. As a result, the creation of environmentally friendly cushioning packaging has become an unavoidable trend [12]. PLA-based and paper-based packaging materials are the most common biodegradable packaging materials on the market today. These two types of materials are expensive and have unsatisfactory cushioning properties [13,14]. In summary, all of these cushioning materials have limitations.

Renewable and biodegradable materials derived from biomass are attractive candidates for replacing non-biodegradable petrochemical plastics. The annual consumption of elastic petroleum-based polymers in the field of packaging and shock absorption is as high as 1.8 million tons, which accounts for about 80% of all non-degradable packaging materials [15]. As a result, we must use bio-based elastic materials to replace petroleum-based elastic materials as soon as possible. Wood is a naturally occurring organic polymer with low density, high modulus, high strength, and biodegradability. Wood-based materials are gaining popularity due to their high output and biodegradability. Wood has received a lot of attention as a natural material that is sustainable, renewable, biodegradable, and environmentally friendly [16]. Wood is highly anisotropic and has a hierarchical structure. Furthermore, it benefits from a diverse range of raw material sources, a high yield, and the ability to be metabolized and broken down by environmental microbes to function as a carbon source in the natural world, eventually converting into inorganic salts and water. As a result, biomass products derived from wood will be critical in the development of green materials.

Nowadays, turning natural wood into soft aerogel has become a hot research topic. Wood aerogel, a lightweight and highly compressible cellulose-based material, is obtained by removing lignin and hemicellulose from natural wood [17,18]. The resulting wood aerogel has a higher porosity than the typical top-down prepared cellulose-based aerogels because of the distinctive hierarchical nanostructure with numerous interconnected arched substructures. Wood aerogels made from natural Balsa are becoming more and more well known for use in solar desalination [19], water treatment [20], and crude oil adsorption [21] due to their distinctive channel configuration. Hu et al. [18] obtained a wood sponge by removing lignin and hemicellulose, which is elastic in the radial direction. This top-down approach is easy and scalable, and is a promising direction for developing high-quality elastic wood directly from wood. In addition, the removal of lignin and hemicellulose leads to an increase in the porosity of the wood, and lays a certain basis for subsequent functionalization. The elastic wood which is obtained by deep delignification and fungal deterioration [17,22,23,24] is easier to functionalize. Through processes such as delignification and loading of reduced graphene oxide (rGO), the elastic wood is turned into a highly sensitive piezoresistive sensors [23,25]. Combined with silane or epoxy modification, hydrophobic wood aerogel sensor, absorbents/filters for a solar-assisted steam generation have been successfully prepared [26,27]. However, some of the distinctive nano-microstructures in the natural wood are removed during this process, and the functional elements are severely removed, leading to the neglect of the natural wood’s functional properties. Additionally, the involved toxic modifiers may have negative environmental effects [28]. Furthermore, lignin and hemicellulose of the wood aerogel have been largely removed, resulting in poor mechanical properties, a lengthy preparation process, and high chemical and material consumption. An anisotropic elastic material can be obtained using a simple delignification and heat treatment process.

Here, Balsa wood has been selected as the primary component in the production of elastic wood due to its abundance and low cost. In addition, it has a thin wood cell wall and a low density, which makes it simple to chemically treat [29,30,31]. In addition, the microstructure of Balsa wood is bimodally porous, three-dimensionally connected, and contains a few smaller vessel channels and much more tracheid-narrowing channels. An elastomer made from natural wood with superhydrophobic and superelastic properties can be prepared using a straightforward chemical and thermal treatment strategy thanks to the low density and thin wood cell walls of the Balsa. Some of the hydrophilic groups are removed by chemical and thermal processes, causing the material acquires super hydrophilicity. The ray structure of the wood opens up to form a leaf-spring-like microstructure when lignin and hemicellulose are removed, which considerably enhances the shape recovery of the elastic wood. As a cushioning material, its excellent shape recovery and resistance provide mechanical support. The removal of the matrix also greatly improves the porosity of the material, laying the groundwork for subsequent functionalization. Herein, loading MWCNT with the elastic wood makes it possible to have electromagnetic. The modified elastic wood not only has excellent mechanical properties, but is also environmentally friendly, giving it a large market potential. A solid foundation has thus been laid for the preparation of various functional cushioning materials.

## 2. Materials and Methods

### 2.1. Materials

Balsa wood (Ochroma lagopus Swartz) was cut into 15 mm cubes (radial × tangential × longitudinal). KOH, Sodium chlorite (NaClO_2_, 80%), Acetic acid, multi-wall carbon nanotube (MWCNT), and Sodium dodecyl benzene sulfonate (SDBS) were purchased from Maclean Biotech Co., Ltd. (Shanghai, China). All chemicals were laboratory grade and were used without further purification. Deionized (DI) water was used as a solvent to treat the wood cubes during the experiments.

### 2.2. Preparation of the Cushion Materials

#### 2.2.1. Preparation of the Elastic Wood

The Balsa cubes were first treated with 2% KOH solution at 90 °C for 6 h to remove hemicellulose. In addition, the cubes were then placed into acetic-acid-buffered sodium chlorite solution (pH = 4–5) and reacted under 80 °C for 10 h to remove lignin. The chemically treated blocks were washed with deionized water until neutral, then freeze-dried for 48 h at −59 °C. The samples were put in a tubular furnace and heated to 300 °C at a heating rate of 4 °C/min and kept for 3 h. After cooling to room temperature, elastic woods were obtained.

#### 2.2.2. Preparation of MWCNT-Embedded Elastic Wood

A 0.5 wt% solution of SDBS was prepared, and 0.05 g of MWCNT was added to it before sonication for 15 min to obtain a well-dispersed solution. To obtain MWCNT-embedded wood, the Balsa cubes were dipped in the MWCNT solution for 30 min, and dried with a hairdryer after taking the cubes out from the solution. This step was repeated three to five times to ensure that the MWCNT was completely embedded into the Balsa. The fully impregnated MWCNT-embedded wood was then placed in a freeze-dryer for 24 h.

### 2.3. Characterizations

#### 2.3.1. Morphological and Chemical Properties

(1) Microscopic morphology

The morphologies of the natural wood and elastic wood were observed using a Hitachi Regulus8100 scanning electron microscope (SEM). All samples were gold coated to improve conductivity. The Balsa wood was cut into samples 10 mm × 10 mm × 5 mm. To improve the flatness of the sample cross-sections, they were rotary cut.

(2) Microscopic pore structure

The prepared material was ground and degassed at 200 °C in a nitrogen atmosphere. Using a physical adsorption apparatus, the material was then subjected to nitrogen adsorption–desorption experiments. Calculation of the pore size distribution of the material was performed according to the Barret Joyner and Halenda (BJH) method [32].

(3) FTIR

The material was ground into a powder and mixed evenly with KBr at a mass ratio of 1:100 before being pressed with a tablet press. The Fourier transform infrared spectroscopy (FTIR) spectra of the samples were examined using a PerkinElmer Frontier in the range of 4000–400 cm^−1^, the resolution was 4 cm^−1^, and 32 scans were performed.

(4) Wood component content testing

The content of lignin was measured according to the standard GB/T 2677.8-94. The contents of holocellulose and cellulose were measured according to the standards GB/T2677.10-1995 and GB/T 744-1989, respectively. Three replicate samples were tested, and the average value was taken.

(5) Raman Spectroscopy

The material was made in powder form. The instrument used was a Horiba LabRAM HR Evolution with a wave number range of 1000–2000 cm^−1^ and a laser wavelength of 532.

#### 2.3.2. Mechanical Properties at Different Temperatures

The mechanical properties of samples were tested with a universal material mechanical machine. The size of the sample to be tested is 15 mm × 15 mm × 15 mm. The material was compressed at a rate of 12 ± 3 mm/min. Multiple compression rates (20%, 40%, 60%) were selected for testing, and the stress–strain curves during compression–rebound were recorded. The number of compression cycles was set to 100 (60% compression rate), and the stress–strain curve was recorded over 100 compression cycles. In this way, the changes in rebound rate and energy loss over the course of 100 cycles of compression were calculated. From this curve, the cushioning coefficient and energy absorption of the material can be calculated, as follows [33]:

(1) $$e=\int_{0}^{\varepsilon }\sigma d\varepsilon =\overset{n}{\underset{i=1}{\sum }}(\sigma_{i}∆\varepsilon_{i}+\frac{1}{2}∆\sigma_{i}∆\varepsilon_{i})$$

and

(2) $$C=\frac{\sigma }{e}$$

where:

*e*—energy absorption per unit volume
*C*—cushioning coefficient
*ε*—strain
*σ*—stress

#### 2.3.3. Finite Element Simulation

Based on the microscopic features of the elastic tangential section, a two-dimensional finite element model containing the leaf-spring-like structure was constructed. Simulations were performed using the commercial finite element software ABAQUS/Standard 6.14. The material was assumed to be isotopically linear-elastic, with Young’s modulus of 0.35 MPa and Poisson’s ratio of 0. Uniaxial compression was applied to the model in the x-direction. Meanwhile, the periodic condition was applied in the y-direction. Frictional contact was defined between all surfaces.

#### 2.3.4. Electromagnetic Shielding Performance

Using the vector network analyzer PNA-N5244A, the material’s electromagnetic shielding performance was evaluated. Samples of the material with dimensions of 15.9 mm × 8.03 mm × 2 mm were manufactured, and subjected to testing at 12.4–18 GHz. Electromagnetic shielding was calculated using the S-parameter with the following formula:

T = |S_21_|^2^

SE_T_ = −10 lgT

where:

S_21_—S-parameter
SE_T_—shielding effectiveness

#### 2.3.5. Drop Test

To simulate the height of the goods during loading and unloading, the beaker drop height was set at 80 cm by ergonomic principles, and a 5 cm × 5 cm × 1.5 cm sample was chosen as a cushioning material. The entire beaker dropping procedure was captured on video. For comparison with the previous group, a control group was created in which the beaker was dropped directly from 80 cm without safety precautions.

## 3. Results and Discussion

### 3.1. Morphology and Structure

**Figure 1:**
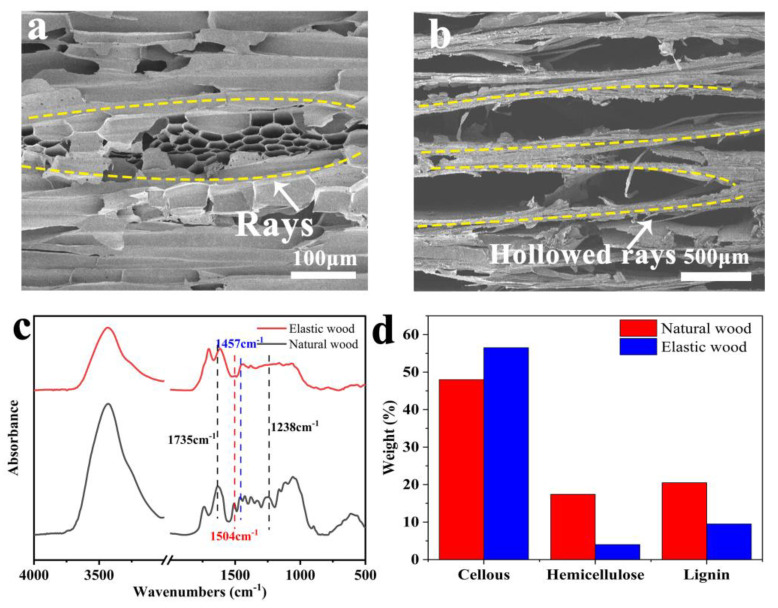
Structure and chemical evolution. (**a**,**b**) SEM images of tangential sections of natural wood and elastic wood. (**c**) FTIR spectra of the different wood samples. (**d**) Relative amounts of cellulose, hemicellulose and lignin in various samples of wood as determined by chemical composition.

The evolution of the structure is shown in the SEM images in Figure 1a,b. More detailed morphological and microstructural changes were also observed by SEM. As shown in Figure 1a,b, the wood cell wall changes from a tightly arranged honeycomb structure to a leaf spring shape after the removal of lignin. This structure governs the material’s elasticity [34]. The formation of this structure is closely linked to the removal of lignin and hemicellulose. In the first step of the KOH pretreatment procedure, some of the hemicellulose is removed, leaving the lignin exposed for later treatment. NaClO_2_ is a widely used oxidizing and bleaching agent that is highly effective in the delignification of wood. When the NaClO_2_ solution is heated, ClO_2_ is produced, and the ClO_2_ oxidizes the phenolic radicals in the lignin molecule to form phenoxy radicals. The lignin molecule is changed into a mucic acid, or the side chain breaks to form a quinone as a result of the subsequent chemical reaction between the generated phenoxy radicals and ClO_2_. Lignin is broken down by oxidation processes, the production of free radicals, chlorate formation, and chlorite conversion with ClO_2_. The partial removal of lignin was further confirmed by the disappearance and shift of the characteristic peaks of lignin at 1504 and 1457 cm^−1^ after chemical treatment by FTIR analysis. The peak relative intensities at 1735 cm^−1^ and 1238 cm^−1^ were shifted and decreased, indicating that hemicellulose was removed, as shown in Figure 1c. In addition to the aforementioned role of KOH, thermal treatment makes a significant contribution to the removal of hemicellulose. Hemicellulose is temperature sensitive; at about 180 °C, the -glucosidic bonds and side chains in the hemicellulose molecular chain break, and esterification takes place between the glyoxylate and -OH groups. All the side chains break, and partial carbonization occurs when the treatment temperature reaches 220 °C. As a result, the thermal treatment causes elastic woods to darken in color and become more hydrophobic [35]. Only the weaker intermolecular bonds, such as β-O-4 and α-O-4, experience C-O breakage when the pyrolysis temperature exceeds 200 °C, because the intermolecular bonds between lignin molecules are generally stable. The pyrolysis of lignin results in the production of CO, CO_2_, and H_2_O. At 200–300 °C, the carboxyl, carbonyl, and ester groups in the side chain of phenyl propane are primarily broken down and converted into CO [36,37,38,39]. It can be seen that the thermal treatment causes the majority of the hemicellulose to go through pyrolysis and only a small amount of the lignin to change, while the cellulose largely stays the same. The subsequent quantitative analysis of the chemical composition also verified the removal of lignin and hemicellulose. As shown in Figure 1d, the lignin content decreases from 20.8% to 9.5%, while the hemicellulose content decreases from 17.6% to 4%.

### 3.2. Mechanical Properties

**Figure 2:**
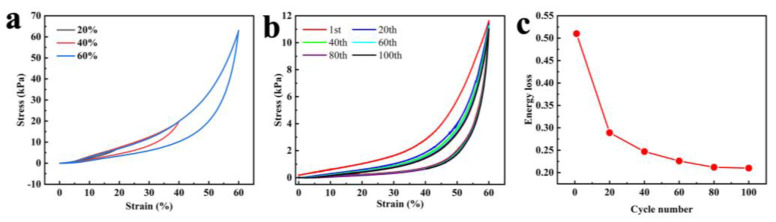
Mechanical compressibility and elasticity of the elastic wood. (**a**) Stress–strain curves of the elastic wood compressed at maximum stress of 20%, 40%, and 60%, respectively. (**b**) Stress–strain curves for the elastic wood under tangential compression for up to 100 cycles of loading and unloading. (**c**) Energy loss of the elastic in different cycles derived from the stress–strain curves in (**b**).

**Figure 3:**
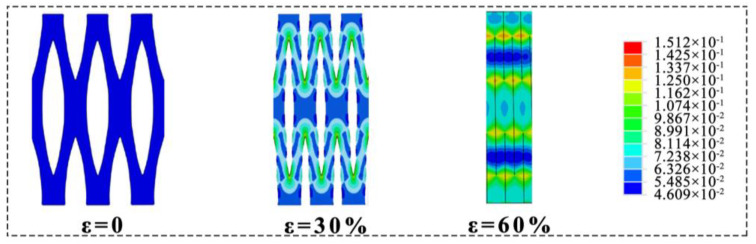
As the compressive strain rises, FEM simulation depicts the evolution of the maximum principle strain distribution.

The removal of lignin and hemicellulose causes the pores between the cell walls to be enlarged, forming the leaf-spring-like microstructure. Because of this structure, the elastic wood retains the anisotropic mechanical properties of natural wood. When a sample is compressed in the longitudinal or radial direction, it is crushed due to the splitting between the cells. As shown in Figure 2a, the material can fully recover up to 60% of the compressive strain in the tangential direction. In addition, the material has excellent fatigue resistance, recovering up to 95% of its original dimensions after 100 cycles of compression at 60% strain with negligible permanent deformation (Figure 2b). The energy loss of the elastic wood is shown in Figure 2c, decreasing from 0.51 in the first cycle to 0.2. This is also relatively low when compared to other aerogel materials [40,41]. Unlike normal petroleum-based elastic materials, the stress–strain in elastic wood has only two stages, yield plateau and densification, and there is no elastic deformation zone. The stress–strain curve shows that at strains greater than 40%, the stress increases sharply. At this point, the pore structure formed by the removal of lignin and hemicellulose is compacted and further compressed. The sharp increase in stress reflects the collision of the laminations with each other during compression. When the stress is withdrawn, the strain decreases to zero, indicating that the volume has been completely recovered without plastic deformation. Finite element simulations (FEM) are used to explain the microscopic mechanism of reversible compression. As shown in Figure 3, when the material is compressed, the stress at the corners of the arch structure is effectively dispersed, resulting in uniform forces on the material and giving it excellent loading properties.

**Figure 4:**
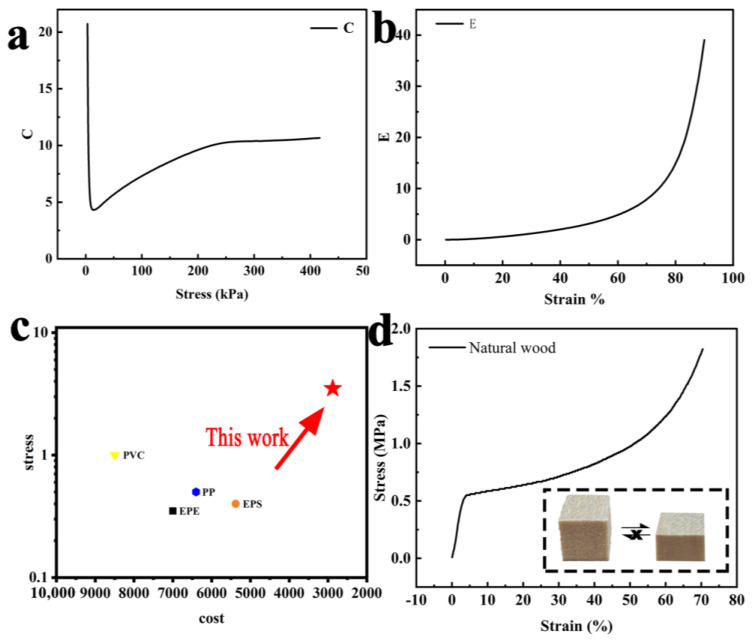
(**a**) Cushioning factor of the elastic wood. (**b**) Energy absorption under different strains. (**c**) Comparison of the mechanical properties and costs of different materials. (**d**) Diagram of natural wood compression and stress–strain curves of the natural wood.

In addition to good elastic recovery and fatigue resistance, this material has a good cushioning performance and energy absorption performance. The cushioning factor is a crucial parameter for determining whether the cushioning packaging is reasonable, and is parameter that reflects the material’s performance in terms of cushioning [33]. Materials with a lower cushioning factor should be chosen when designing cushioning packaging in order to minimize the amount of cushioning material. The cushioning properties of a material are usually negatively correlated with its hardness, with poor elasticity and cushioning properties being observed when the prepared material is hard. The material obtained by chemical and thermal treatment is an elastic material with excellent mechanical properties and has a cushioning factor of 4.3 (Figure 4a), which gives it good cushioning properties, comparable to those of commonly used plastic cushioning materials [42]. The internal pores of the wood were enlarged after delignification, and the resulting holes are the first to bear pressure during compression [43]. The energy absorption per unit volume of elastic wood increases as stress increases gradually [44], as shown in Figure 4b. For comparison, compression tests on natural wood were performed, and it was discovered that the natural wood had a strain recovery rate of zero, as shown in Figure 4d. The natural wood has a high resistance to compression because it has never been chemically treated. The stress–strain curve is depicted in Figure 4d, and it can be seen that at strains of 0–5%, the material’s elastic deformation zone, the stress increases sharply to 500 kPa; at strains of 5–50%, the stress increases slowly; and when the stress value exceeds 50%, the stress value increases sharply. The ability of the materials used in cushioning packaging to effectively absorb energy during compression is a key characteristic. By contrast, natural wood only produces a trace of compression and requires high stresses, which limits its potential for use in cushioning packaging. In addition to good mechanical properties, excellent fatigue resistance combined with low cushioning coefficients and high absorption energy per unit volume, the elastic wood also offers lower costs, as shown in Figure 4c, which shows better mechanical properties combined with lower costs compared to conventional cushioning materials. These advantages make the elastic wood a promising alternative to petroleum-based cushioning materials.

### 3.3. Electromagnetic Shielding Performance

**Figure 5:**
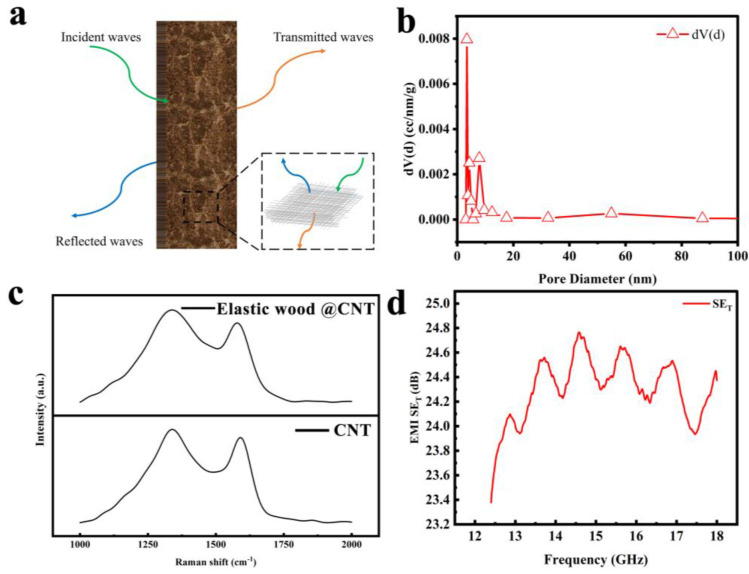
(**a**) The electromagnetic shielding mechanism diagram. (**b**) Distribution of pores in elastic wood. (**c**) Raman spectra of MWCNT-embedded elastic wood. (**d**) The electromagnetic shielding effectiveness of the material.

The combination of intrinsic channels and elasticity has a wide range of applications. The porosity of the samples is increased by chemical and thermal treatment, allowing them to be loaded with other functional materials. The pore size distribution of the elastic wood is shown in Figure 5b. Pores about 10 nm in size primarily predominate in the elastic wood. A 0.05 wt% solution of multi-walled carbon nanotubes was prepared using sodium dodecyl sulfate solution as the dispersion. The elastic wood gained in weight by 5.7% before and after vacuum impregnation, indicating that CNT was attached to the elastic wood. This was followed up with further Raman spectroscopic analysis of elastic wood@CNT. It is well known that the Raman spectra of CNT exhibit a G-band associated with tangential vibrational modes around 1582 cm^−1^, while the D-band around 1350 cm^−1^ originates from disorder or defects [45]. Based on this, the loading of MWCNT can be confirmed by examining the G-band of the Raman spectra measured from each sample. From the reference spectra shown in Figure 5c, we can see that the two main spectra at 1338.1 cm^−1^ correspond to the D-band, while the vibrational modes at 1577.3 cm^−1^ correspond to the G-band. This further confirms the payload of the CNT. The waveguide method is generally used to test the electromagnetic shielding effectiveness of block materials, and the shielding principle of the material is shown in Figure 5a. The material was tested for electromagnetic shielding effectiveness using a vector network analyzer and the results are shown below. Figure 5d shows that the material has excellent electromagnetic shielding properties, which protect the packaged product from electromagnetic interference or preventing it from emitting electromagnetic waves into the surrounding environment, when the product contains electromagnetic radiation. The new material, a composite of elastic wood and multi-walled carbon nanotubes, combines the respective advantages of both materials, maintaining the excellent mechanical properties of elastic wood while effectively suppressing the various electromagnetic waves propagating in space and the resulting electromagnetic interference, ensuring the safety of the human body and information.

### 3.4. Temperature-Invariant Mechanical Properties and Impact Resistance

**Figure 6:**
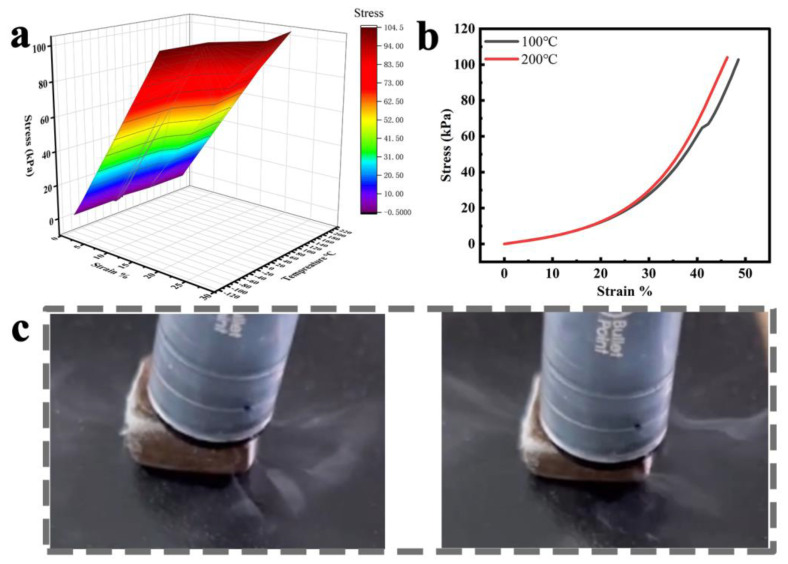
(**a**) 3D surface graphs of the stress dependence on strain and temperature in the compression processes of the elastic wood, exhibiting clear temperature invariance from −100 to 200 °C. (**b**) Stress–strain curves at 100 °C and 200 °C. (**c**) Schematic representation of the compression process after immersion in liquid nitrogen.

As a cushioning packaging material, it may be necessary for the material to have a low sensitivity to temperature, thus helping to protect goods from external temperature changes. Because of its unique cellulose backbone structure, the elastic wood can withstand extreme low and high temperatures. Five temperatures ranging from −100 °C to 200 °C were chosen for stress–strain testing. The results are presented in the form of a colored 3D surface, demonstrating that stresses remained essentially constant across a wide temperature range during compression (Figure 6a). The stress–strain curves for elastic wood at 100 °C and 200 °C largely overlap, as shown in the stress–strain curve graph in Figure 6b. In addition to the high-temperature environment, we also compressed the elastic wood in liquid nitrogen, as shown in video S1. The liquid nitrogen-soaked blocks were then removed and compressed, as shown in Figure 6c, demonstrating that the blocks retain compressive resilience after being soaked in liquid nitrogen. This further demonstrates the mechanical stability of the elastic wood over a wide temperature range.

**Figure 7:**
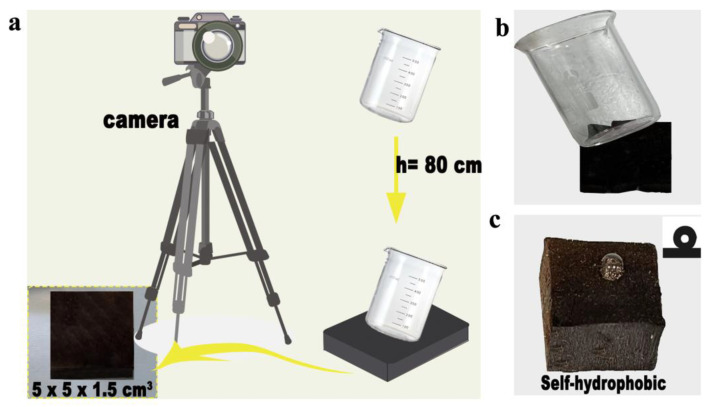
(**a**,**b**) Drop test to simulate the loading and unloading process. (**c**) Wettability of the elastic wood.

As a cushioning material, the most important aspect is to protect the product; therefore, we performed a simple drop test in the laboratory and set the drop height to 80 cm according to ergonomics, imitating the height of the product during loading and unloading. The beaker was dropped from a height of 80 cm, and the entire process was recorded with a video camera, as shown in Figure 7a,b. As can be seen in videos S2 and S3, the beaker without the elastic wood as a cushioning liner shattered immediately upon landing, whereas the beaker with the elastic wood as a cushioning liner bounced off the ground, remaining intact, further confirming the usability of elastic wood as a cushioning material. Furthermore, hydrophilic groups such as hydroxyl groups are removed during the thermal treatment, allowing the elastic wood to acquire a degree of self-hydrophobicity with a hydrophobic angle of 152° (Figure 7c). The cushioning material’s hydrophobic effect also helps to protect the product to some extent. This safeguards the product against the effects of water under adverse storage and transportation conditions such as rain or humidity.

## 4. Conclusions

We developed a wood elastomer through a simple chemical and thermal treatment that has the potential for multifunctional development with a wide temperature range of superelasticity, self-hydrophobicity, and high porosity. The removal of lignin and the thermal treatment process resulted in the thinning of the cell walls of the wood and the formation of an arch-shaped spring structure that became elastic. The results showed that the spring microstructure significantly improved the mechanical properties in the tangential direction, resulting in a strain recovery rate of up to 90% and fatigue resistance in the tangential direction (99% recovery stress retention after 100 compression cycles). It also expands the possibilities for its use in cushioning materials. The porosity of the elastic wood greatly improves its loading and impregnation properties, and the addition of CNT allows the elastic wood to retain its excellent mechanical properties while gaining electromagnetic shielding properties. Because of its unique physicochemical properties, elastic wood has a wide range of promising applications in functional and cushioning packaging.

## Data Availability

All the data of this is included in the manuscript and supplementary material.

## Supplementary Materials

The following supporting information can be downloaded at: https://www.mdpi.com/article/10.3390/polym15061417/s1, Videos S1–S3.
